# Persistent Postural Perceptual Dizziness in Episodic Vestibular Disorders

**DOI:** 10.3390/audiolres12060058

**Published:** 2022-10-27

**Authors:** Valeria Gambacorta, Alessandra D’Orazio, Vincenzo Pugliese, Alfredo Di Giovanni, Giampietro Ricci, Mario Faralli

**Affiliations:** Department of Surgical and Biomedical Sciences, Section of Otorhinolaryngology, University of Perugia, 06129 Perugia, Italy

**Keywords:** dizziness, precipitating conditions, episodic vestibular disorder

## Abstract

Benign Paroxysmal Positional Vertigo (BPPV), Vestibular Migraine (VM), and Meniere Disease (MD) are among the most common episodic vestibulopathies. Persistent Postural Perceptual Dizziness (PPPD) is a chronic functional vestibular disorder that can arise in patients suffering from one or more of these conditions. We analyzed the role of these vestibular disorders as single or multiple associated comorbidities and as a precipitating condition for PPPD. A total of 376 patients suffering from dizziness with a known history of single or multiple vestibular disorders were preliminarily evaluated. We conducted a careful anamnesis to determine whether the reported dizziness could meet the diagnostic criteria for PPPD. PPPD was diagnosed in 24 cases; its incidence in patients with history of a single comorbidity or multiple vestibular comorbidities was 3.9% and 22.4%, respectively. BPPV, VM, and MD were identified as a precipitating condition in 2.34%, 16.45%, and 3.92%, respectively. BPPV constituted a precipitating condition mainly at the first episode. We observed that the presence of multiple vestibular comorbidities (BPPV, VM, and MD) in patients’ clinical history increased the risk of PPPD. VM plays a significant role in representing a precipitating condition for PPPD, both when present individually or in association with the other vestibular disorders.

## 1. Introduction

Persistent postural perceptual dizziness (PPPD) is classified as a chronic functional vestibular disorder; it is not a psychiatric or structural condition, even though both can coexist with the disease. The Barany society formulated diagnostic criteria for PPPD in a consensus paper in 2017 [1]. PPPD is a relatively new nosological entity, but it groups characteristics common to other syndromes such as phobic postural vertigo [2], space motion discomfort [3], visual vertigo [4], and subjective chronic dizziness [5]. PPPD presents with one or more symptoms of non-spinning vertigo, unsteadiness or dizziness that arise on most days for three months or more and can worsen with active or passive movements, upright posture, and exposure to moving or complex visual stimuli [1]. Possible pathophysiologic processes underlying PPPD have been identified, which include functional changes in postural control strategies [6,7], changes in the processing of multisensory information [8], and less central integration of threat assessment and spatial orientation networks [9]. The diagnostic criteria also specify that PPPD is usually triggered by circumstances that interfere with equilibrium or cause vertigo, unsteadiness, or dizziness, including vestibular disease of both central and peripheral type, other medical conditions, or psychological distress [5,10]. Most of the disorders that occur before the onset of PPPD can be acute or episodic, and patients report the onset of chronic PPPD symptoms as a result of their acute illness [1]. Vestibular neuritis is a frequent precipitating factor among the causes of isolated vertigo; on the other hand, benign paroxysmal positional vertigo (BPPV), vestibular migraine (VM), and Meniere’s disease (MD) represent the episodic vestibular disorders that most commonly can constitute precipitating events. Considering the comorbidity between migraines and MD [11,12] and the possibility of an overlap syndrome between VM and MD [13], on the one hand, and the high prevalence of BPPV in migraines [14] with or without VM, on the other, it is possible that one or more of these conditions are present in the same patient. Consequently, PPPD can also arise in patients suffering from one or more of these vestibular disorders. The aim of the study was to analyze the role of these vestibular diseases as single or variously associated comorbidities and as a precipitating condition for PPPD.

## 2. Materials and Methods

A consecutive series of patients suffering from a dizziness condition, defined according to the international criteria [15], was preliminarily evaluated. They came to our observation in the period from 5 January 2018 to 31 January 2022. For the purposes of our investigation, we considered patients suffering from dizziness with a history of BPPV [16], VM [17], and MD [18]. In this regard, the patients were divided into two groups according to the presence in anamnesis of a single (BPPV or VM or MD) or several variously associated vestibular disorders (BPPV + VM, BPPV + MD, VM + MD, or BPPV + VM + MD). In these cases, the vestibulopathies were considered comorbidities and did not necessarily represent a precipitating condition for PPPD. In the BPPV, patients with one or more than one episode in their history were considered. Among these, we excluded patients who presented, at the time of the enrollment in this study, the typical signs and symptoms of the vertigo attacks (acute phase) that characterized the aforementioned vestibular disorders. In the same way, patients with VM who presented attacks in the three months before the investigation were excluded. A careful medical history was conducted in all patients to determine whether the reported dizziness could meet the PPPD diagnostic criteria issued by the Barany Society [1]. The overall incidence of PPPD in the population examined and the relative incidence among the vestibular disorders considered were calculated, both as a single comorbidity or multiple comorbidities and as a precipitating condition. The triggering role of these vestibulopathies was analyzed through the recognition of precipitating conditions that are identified in one of the typical attacks of BPPV, VM, and MD. Statistical evaluation of the percentages of incidence was performed by means of the chi-square test. The statistical significance was set for *p* values < 0.05.

## 3. Results

In total, 502 consecutive patients suffering from dizziness were preliminarily examined. Of these, 376 patients had a known history of episodic vertigo and were, therefore, definitively recruited. In particular, 327 were affected by a single vestibular disorder (BPPV, VM or MD), while 49 patients presented variously associated vestibular comorbidities. The distribution of the vestibular disorders among the 376 patients considered is shown in Table 1.

Of the 298 patients with documented BPPV in their history, 83 had a single episode, while 215 had two or more. PPPD was diagnosed, according to the Barany Society criteria, in 24 (6.4%) of the 376 patients examined. In Table 1, we show the distribution of PPPD within the vestibular disorders considered in the study. The incidence of PPPD in patients with a history of BPPV, either isolated or associated with VM and/or MD, was 4.36% (13 PPPD/298 BPPV). In patients with VM and MD, isolated or variously associated with each other and/or with BPPV, there was an incidence of PPPD of 19% (15 PPPD/79 VM) and 15.7% (8 PPPD/51 MD), respectively. Therefore, the incidence of PPPD was significantly higher in patients with a history of VM and/or MD than in those with BPPV (*p* < 0.05). There was no statistically significant difference in the incidence of PPPD between patients with a history of VM or MD (*p* > 0.05). In patients with more vestibular comorbidities, there was a PPPD incidence of 22.4%, significantly higher (*p* < 0.05) than the incidence values in patients with a history of a single vestibular disorder (3.9%) (Table 2).

BPPV was identified as a precipitating condition in 7 of the 24 people diagnosed with PPPD. VM and MD represented, on the other hand, a precipitating condition in 13 and 2, respectively. In two cases, the precipitating condition was identified as an extravestibular cause (atrial fibrillation and head trauma) (Table 3).

The incidence of BPPV, VM, and MD as a precipitating condition was 2.34% (7 of 298), 16.45% (13 of 79), and 3.92% (2 of 51), respectively. There was a significant statistical increase in the incidence as a precipitating factor in patients with VM compared to MD (*p* = 0.028) and especially compared to BPPV (*p* = 0.00001). No statistical difference emerged between patients with BPPV or MD (*p* > 0.05). Lastly, the incidence of BPPV as a precipitating condition was significantly higher (*p* = 0.009) in patients with a single episode (6%) compared to those with two or more episodes in their life (0.9%) (Table 4).

## 4. Discussion

The definition of PPPD underlines the importance of precipitating events that initiate the syndrome. These include all the morbid, vestibular and extravestibular conditions, capable of causing a dizzy condition: isolated or recurrent or chronic exacerbated vestibulopathy, atrial fibrillation, dysautonomic syndromes, brain trauma, and panic attacks [5,10]. The study includes only the most common episodic vestibular disorders; therefore, a frequent precipitant such as vestibular neuritis or other non-vestibular causes have not been considered. Our study confirmed that PPPD may be preceded by episodic vestibular diseases such as VM, MD, and BPPV. These conditions are acute in nature, and the characteristic symptoms of each episodic disorder are distinct, in contrast to the persistent unsteadiness, dizziness and non-spinning vertigo that are the clinical markers of PPPD. In the present study, the role of BPPV, MV, and MD as single or variously associated comorbidities and as a precipitating condition for PPPD was evaluated. In the first case, a vestibular disorder is meant as a disease capable of possibly inducing the syndrome without necessarily being the precipitating condition. In the second case, the vestibular disorder is identified in the vertigo attack capable of constituting the initial event that triggers the PPPD. The study shows that the presence of multiple vestibular comorbidities in patients clinical history increase the risk of PPPD. Among these, VM and MD were the episodic vestibular disorders most represented, while BPPV was documented at a significantly lower percentage. Although dizziness was largely represented in patients with a history of BPPV apart from vertigo attacks, only in a low percentage of cases was the diagnostic criteria for PPPD fulfilled. Residual dizziness has been described in the literature as a symptom that often follows resolution of a BPPV after repositioning maneuvers [19]. Several pathogenetic mechanisms have been hypothesized for this condition, and a considerable variability in its duration is described, from days to weeks [20,21]. In many of these cases, resolution of vertigo is likely to occur within three months of the acute BPPV episode [20]. Therefore, the time criterion required for the diagnostic definition of a PPPD would be lost. In addition to this, residual dizziness after resolution of the BPPV would present, in many cases, qualitative aspects different from those required by the diagnostic criteria for PPPD. Instead of postural dizziness exacerbated by standing and exposure to moving or complex stimuli, there may be a prevailing dizziness in a supine position and positional induced (without vertigo and nystagmus). In a previous study, it was found that only 12.5% of patients with residual dizziness after BPPV resolution subsequently developed PPPD [22]. Even if vestibulopathies are considered as conditions that precipitate PPPD, once again, a lower incidence of BPPV was noted compared to VM. It is interesting to note that PPPD predominantly occurs in patients who have suffered from the first episode of BPPV. This can represent a sensation never experienced by the patient, with an important emotional impact. In recurrent BPPV, in contrast, the patient no longer has the surprise effect associated with the first episode, being accustomed to the recurrence of vertigo. Additionally, since the patient knows the symptom and its symptomatic manifestation, he avoids critical situations, such as cephalic movements that trigger vertigo. In other words, patients exhibit self-control with recurrent BPPV compared to those at the first episode and other episodic vestibular disorders, in which there is no protective mechanism in place, resulting in unpredictable and uncontrollable attacks. In this sense, VM is less controllable by the patients due to its pleomorphism. This, inevitably, has different emotional repercussions. In the literature it is known that, among the most studied vestibular pathologies, patients with BPPV have lower levels of anxiety than those with VM and MD [23]. In these cases, additional symptoms such as headache in VM and tinnitus, fullness, and hearing loss in MD [18], play a significant role. The most relevant data of the present study are expressed by the significant role of VM in representing a precipitating condition for PPPD, both when present individually or in association with the other vestibular disorders. These data are supported by a recent paper, which showed that VM represented a precipitating condition in 25% of cases, the most common among vestibular disorders [24]. Referring to the historical context that led to the definition of PPPD, the literature highlights two aspects: visual addiction and anxiety. Already in 1986 Brandt defined Phobic Postural Vertigo as a syndrome which, in subjects with compulsive-obsessive personality traits, is characterized by: anxiety, fluctuating instability and postural dizziness [2]. Bronstein observed the symptom of Visual Vertigo in a part of patients in his clinical practice [4]. Subjects with Visual Vertigo exhibited visual dependence, a tendency to rely on visual information for spatial orientation. Both of the factors we have discussed can be favored by the mechanisms of migraine. The interaction between migraine and anxiety networks has been documented. The hypothesis is that the increased release of neurotransmitters such as dopamine, norepinephrine, 5-hydroxytryptophan, in addition to disturbances of the calcium channels in the brain and inner ear, are responsible for the symptoms that arise during a migraine attack, such as vertigo and headache [25]. These are the same central neurotransmitters in the processes of anxiety and depression [26]. Furthermore, among the symptoms indicated in the diagnostic criteria of VM, visually induced vertigo has a significant impact in terms of frequency and symptomatology, even considering the high pleomorphism of this pathology [17,27]. A core of symptoms is common between VM and PPPD. For example, VM patients have an increased sensitivity to self-motion or motion of objects in the surroundings and have difficulty performing tests that require precise visual focus. Furthermore, there are VM patients who may take four weeks to fully recover from an episode [17]. For this reason, it can be quite difficult to distinguish the two conditions. However, the temporal data defined by the diagnostic criteria can facilitate the diagnosis. In fact, symptoms in PPPD must occur on most days for three months or more. Most of those affected report symptoms every day or almost every day [1]. Therefore, the tendency to a greater chronicity of symptoms indicates a more probable diagnosis of PPPD. In addition, the patients recruited in the study had not presented crises in the last three months, demonstrating good control of migraine mechanisms in the strict sense. These data consolidate the temporal criterion that defines the diagnosis of PPPD. The last data from our study to consider are the different behaviors that emerged between VM and MD in terms of comorbidity or a precipitating condition for PPPD. Although MD represents, as does VM, an important comorbidity in a population of patients with episodic vestibular disorders, it does not show the same ability to constitute a precipitating condition. The reason may lie in a more extensive involvement of central nervous system during VM attacks or a lower degree of visual dependence arising from MD. Both reasons could make VM more prone to represent a condition that precipitates PPPD in patients with a history of single or multiple episodic vestibular disorders.

## 5. Conclusions

Our study confirmed that PPPD may be preceded by episodic vestibular disorders such as VM, MD, and BPPV. The study showed that the presence of multiple vestibular comorbidities in patients’ clinical history increased the risk of PPPD. Among these, VM and MD were the episodic vestibular disorders most represented. The most relevant data of the study were expressed by the significant role of VM in representing a precipitating condition for PPPD, both when present individually or in association with other vestibular disorders. PPPD mainly involved patients who had the first episode of BPPV.

## Figures and Tables

**Table 1:** Distribution of the episodic vestibular disorders (single or multiple) and Persistent Postural Perceptual Dizziness in the clinical history of the 376 patients recruited. Persistent Postural Perceptual Dizziness: PPPD. Benign Paroxysmal Positional Vertigo: BPPV. Vestibular Migraine: VM. Meniere Disease: MD.

	Single Vestibular Disorder				Multiple Vestibular Disorders				
Vestibular Disorder	BPPV	VM	MD	Total	BPPV, VM	BPPV, MD	VM, MD	BPPV, VM, MD	Total
n° patients	257	46	24	327	22	16	8	3	49
n° PPPD	5	6	2	13	5	2	3	1	11

**Table 2:** Incidence (%) of the Persistent Postural Perceptual Dizziness in the episodic vestibular disorders considered as comorbidities. Statistical evaluation and comparison between the groups. Persistent Postural Perceptual Dizziness: PPPD. Benign Paroxysmal Positional Vertigo: BPPV. Vestibular Migraine: VM. Meniere Disease: MD. Single Vestibular Disorder: SVD. Multiple Vestibular Disorders: MVD.

	% PPPD in BPPV	% PPPD in VM	% PPPD in MD	% PPPD in SVD	% PPPD in MVD	Chi Square Statistic Value	*p* Value
BPPV vs. VM	4.36	19				19.4262	0.00001
BPPV vs. MD	4.36		15.7			9.8743	0.016
VM vs. MD		19	15.7			0.2319	0.6
SVD vs. MVD				3.9	22.4	24.3374	0.00001

**Table 3:** Distribution of the precipitating condition. Persistent Postural Perceptual Dizziness was diagnosed in 24 cases among the 376 patients recruited with clinical history of single or multiple episodic vestibular disorders. Persistent Postural Perceptual Dizziness: PPPD. Benign Paroxysmal Positional Vertigo: BPPV. Single (or first) episode of Benign Paroxysmal Positional Vertigo: BPPV (1). More than one episode (or not the first) of Benign Paroxysmal Positional Vertigo: BPPV (>1). Vestibular Migraine: VM. Meniere Disease: MD. Head trauma: HT. Atrial Fibrillation: AF.

Vestibular Disorders	BPPV	VM	MD	BPPV + VM	BPPV + MD	VM + MD	BPPV + VM + MD
PPPD	5	6	2	5	2	3	1
Precipitating Condition	3 BPPV (1) 1 HT 1 AF	6 VM	2 MD	1 BPPV (1) 4 VM	1 BPPV (1) 1 BPPV (>1)	3 VM	1 BPPV (>1)

**Table 4:** Incidence (%) of the Persistent Postural Perceptual Dizziness in the episodic vestibular disorders considered as precipitating conditions. Statistical evaluation and comparison between the groups. Persistent Postural Perceptual Dizziness: PPPD. Benign Paroxysmal Positional Vertigo: BPPV. Single (or first) episode of Benign Paroxysmal Positional Vertigo: BPPV (1). More than one episode (or not the first) of Benign Paroxysmal Positional Vertigo: BPPV (>1). Vestibular Migraine: VM. Meniere Disease: MD.

	% PPPD in BPPV	% PPPD in VM	% PPPD in MD	% PPPD in BPPV (1)	% PPPD in BPPV (>1)	Chi Square Statistic Value	*p* Value
BPPV vs. VM	2.34	16.5				24.7365	0.00001
BPPV vs. MD	2.34		3.92			0.4287	0.512
VM vs. MD		16.5	3.92			4.7702	0.02
BPPV (1)vs.BPPV (>1)				6	0.9	6.7739	0.009

## Data Availability

The data presented in this study are available on request from the corresponding author.

## References

[1] Staab J.P., Eckhardt-Henn A., Horii A., Jacob R., Strupp M., Brandt T., Bronstein A. (2017). Diagnostic criteria for persistent postural-perceptual dizziness (PPPD): Consensus document of the committe for the classification of the vestibular disorders of the Barany Society. J. Vestib. Res..

[2] Brandt T. (1996). Phobic postural vertigo. Neurology.

[3] Jacob R.G., Redfern M.S., Furman J.M. (2009). Space and motion discomfort and abnormal balance control in patients with anxiety disorders. J. Neurol. Neurosurg. Psychiatry.

[4] Bronstein A.M. (1995). Visual vertigo syndrome: Clinical and posturographyfindings. J. Neurol. Neurosurg. Psychiatry.

[5] Staab J.P., Ruckenstein M.J. (2007). Expanding the differential diagnosis of dizziness. Arch. Otolaryngol. Head Neck Surg..

[6] Holmberg J., Tjernström F., Karlberg M., Fransson P.A., Magnusson M. (2009). Reduced postural differences between phobic postural vertigo patients and healthy subjects during a postural threat. J. Neurol..

[7] Schniepp R., Wuehr M., Huth S., Pradhan C., Brandt T., Jahn K. (2014). Gaits characteristics of patients with phobic postural vertigo: Effects of fear of falling, attention, and visual input. J. Neurol..

[8] Cousins S., Kaski D., Cutfield N., Arshad Q., Ahmad H., Gresty M.A., Seemungal B.M., Golding J., Bronstein A.M. (2017). Predictors of clinical recovery from vestibular neuritis: A prospective study. Ann. Clin. Transl. Neurol..

[9] Indovina I., Riccelli R., Chiarella G., Petrolo C., Augimeri A., Giofrè L., Lacquaniti F., Staab J.P., Passamonti L. (2015). Role of the insula and vestibular system in patients with chronic subjective dizziness: An fMRI study using sound-evoked vestibular stimulation. Front. Behav. Neurosci..

[10] Staab J., Eggers S., Neff B., Shepard N., Goulson A., Carlson M. (2010). Validation of a clinical syndrome of persistent dizziness and unsteadiness. Abstracts from the XXVI Barany Society Meeting, Reykjavik, Iceland, August 18-21, 2010. J. Vest. Res..

[11] Radtke Lempert T., Gresty M.A., Brookes G.B., Bronstein A.M., Neuhauser H. (2002). Migraine and Menière’s disease: Is there a link?. Neurology.

[12] Gopen Q., Virre E., Anderson J. (2009). Epidemiologic study to explore links between Ménière syndrome and migraine headache. Ear Nose Throat J..

[13] Murofushi T., Tsubota M., Kitao K., Yoshimura E. (2018). Simultaneous Presentation of Definite Vestibular Migraine and Definite Ménière’s Disease: Overlapping Syndrome of Two Diseases. Front. Neurol..

[14] Kim S.K., Hong S.M., Park I.S., Choi H.G. (2019). Association Between Migraine and Benign Paroxysmal Positional Vertigo Among Adults in South Korea. JAMA Otolaryngol. Head NeckSurg..

[15] Barany Society Initiative for the Establishment of the International Classification of Vestibular Disorders (ICVD). http://www.jvr-web.org/images/InstructionsforICVDsubcommitteesasof19Oct2014.pdf.

[16] von Brevern M., Bertholon P., Brandt T., Fife T., Imai T., Nuti D., Newman-Toker D. (2015). Benign paroxysmal positional vertigo: Diagnostic criteria. Consensus document of the Committee for the Classification of Vestibular Disorders of the Barany Society. J. Vestib. Res..

[17] Lempert T., Olesen J., Furman J., Waterston J., Seemungal B., Carey J., Bisdorff A., Versino M., Eversi S., Newman-Toker D. (2012). Vestibular migraine: Diagnostic criteria. Consensus document of the Barany Society and the International Headache Society. J. Vestib. Res..

[18] Escamez J.A.L., Carey J., Chung W.H., Goebel J.A., Magnusson M., Mandala M. (2015). Diagnostic criteria for Meniere’s Disease. Consensus document of the Barany Society, The Japan Society for Equilibrium Research, the European Academy of Otology and Neurotology (EAONO), the American Academy of Otolaryngology-Head and Neck Surgery (AAO-HNS) and the Korean Balance Society. J. Vestib. Res..

[19] Lee N.H., Kwon H.J., Ban J.H. (2008). Analysis of residual symptoms after treatment in benign paroxysmal positional vertigo using questionnaire. Otolaryngol. Head NeckSurg..

[20] Teggi R., Quaglieri S., Gatti O., Benazzo M. (2013). Residual dizziness after successful repositioning manuevers for idiopathic benign paroxysmal positional vertigo. ORL J. Otorhinolaryngol. Relat Spec..

[21] Faralli M., Lapenna R., Giommetti G., Pellegrino C., Ricci G. (2016). Residual dizziness after the first BPPV episode: Role of otolithic function and of a delayed diagnosis. Eur. Arch. Otorhinolaryngol..

[22] Tropiano P., Lacerenza L.M., Agostini G., Barboni A., Faralli M. (2021). Persistent postural perceptual dizziness following paroxysmal positional vertigo in migraine. Acta Otorhinolaryngol. Ital..

[23] Yuan Q., Yu L., Shi D., Ke X., Zhang H. (2015). Anxiety and depression among patients with different types of vestibular peripheral vertigo. Medicine.

[24] Watersto J., Chen L., Mahony K., Gencarelli J., Stuart G. (2021). Persistent Postural-Perceptual Dizziness: Precipitating Conditions, Co-morbidities and Treatment With Cognitive Behavioral Therapy. Front. Neurol..

[25] Cutrer F.M., Baloh R.W. (1992). Migraine-associated dizziness. Headache.

[26] Balaban C.D., Jacob R.G., Furman J.M. (2011). Neurologicbases for comorbidity of balance disorders, anxiety disorders and migraine: Neurotherapeutic implications. Expert Rev. Neurotherap..

[27] Ori M., Arra G., Caricato M., Freccia R., Frati F., De Bonis T., Ricci G., Faralli M. (2020). Age-related features in vestibular migraine onset: A multiparametric analysis. Cephalalgia.

